# Comparison of simultaneous auscultation and ultrasound for clinical assessment of bowel peristalsis in neonates

**DOI:** 10.3389/fped.2023.1173332

**Published:** 2023-09-19

**Authors:** Archana Priyadarshi, Mark Tracy, Pankhuri Kothari, Chiranjibi Sitaula, Murray Hinder, Faezeh Marzbanrad, Stephanie Morakeas, Amit Trivedi, Nadia Badawi, Sheryl Rogerson

**Affiliations:** ^1^ Department of Neonatology, Westmead Hospital Neonatal Intensive Care Unit, Sydney, NSW, Australia; ^2^ Grace Neonatal Intensive Care Unit, The Children’s Hospital Westmead, Sydney, NSW, Australia; ^3^ Department of Electrical & Computer Systems Engineering, Monash University, Clayton, VIC, Australia; ^4^ Department of Neonatal Intensive Care Unit, The Royal Women’s Hospital, Melbourne, VIC, Australia

**Keywords:** neonatal bowel sounds, bowel peristalsis, abdominal ultrasound, auscultation, neonates

## Abstract

**Introduction:**

Assessment of bowel health in ill preterm infants is essential to prevent and diagnose early potentially life-threatening intestinal conditions such as necrotizing enterocolitis. Auscultation of bowel sounds helps assess peristalsis and is an essential component of this assessment.

**Aim:**

We aim to compare conventional bowel sound auscultation using acoustic recordings from an electronic stethoscope to real-time bowel motility visualized on point-of-care bowel ultrasound (US) in neonates with no known bowel disease.

**Methods:**

This is a prospective observational cohort study in neonates on full enteral feeds with no known bowel disease. A 3M™ Littmann® Model 3200 electronic stethoscope was used to obtain a continuous 60-s recording of bowel sounds at a set region over the abdomen, with a concurrent recording of US using a 12l high-frequency Linear probe. The bowel sounds heard by the first investigator using the stethoscope were contemporaneously transferred for a computerized assessment of their electronic waveforms. The second investigator, blinded to the auscultation findings, obtained bowel US images using a 12l Linear US probe. All recordings were analyzed for bowel peristalsis (duration in seconds) by each of the two methods.

**Results:**

We recruited 30 neonates (gestational age range 27–43 weeks) on full enteral feeds with no known bowel disease. The detection of bowel peristalsis (duration in seconds) by both methods (acoustic and US) was reported as a percentage of the total recording time for each participant. Comparing the time segments of bowel sound detection by digital stethoscope recording to that of the visual detection of bowel movements in US revealed a median time of peristalsis with US of 58%, compared to 88.3% with acoustic assessment (*p* < 0.002). The median regression difference was 26.7% [95% confidence interval (CI) 5%–48%], demonstrating no correlation between the two methods.

**Conclusion:**

Our study demonstrates disconcordance between the detection of bowel sounds by auscultation and the detection of bowel motility in real time using US in neonates on full enteral feeds and with no known bowel disease. Better innovative methods using artificial intelligence to characterize bowel sounds, integrating acoustic mapping with sonographic detection of bowel peristalsis, will allow us to develop continuous neonatal bowel sound monitoring devices.

## Introduction

Peristalsis (bowel motility) is an inherent function of a healthy bowel. Bowel sounds heard via stethoscopes have been a traditional means of assessment of bowel activity, and they have thus been presumed to represent peristalsis. These sounds reflect movements of luminal content in different parts of the gut. The precise mechanisms of these sounds are not clear, i.e., how muscular peristalsis behaves with solid, liquid, and gas components luminally.

Bowel sound assessment is part of the general abdominal examination and involves:

1. Placing a stethoscope over the abdominal wall.
2. Manually counting the number of times bowel sounds are heard over at least 1 min (ideally over 3 min), usually in one specified location over the abdominal wall. The recommended assessment area is the right iliac fossa.
3. Categorizing bowel sounds heard by the operator as absent, present, or diminished (1–3).

To screen for bowel pathology, neonatal clinicians rely on the detection of bowel sounds by auscultation, clinical examination findings, clinical information such as evidence of any gastric milk residue, and description and timing of stools passed. Sometimes a plain abdominal radiograph screen is necessary to evaluate for any abnormality in the bowel gas pattern. Reduced bowel sounds or absence of sounds over a brief examination period may reflect serious systemic pathology, such as sepsis, and/or localized NEC. In addition, acidosis can impair bowel wall perfusion and contractility of the bowel. Alterations in intestinal neuronal cell migration and gut microbiome can also lead to gut motility disorders and feed intolerance.

This assessment screening for bowel pathology is a common clinical scenario in the management of extremely preterm infants on high-level respiratory support using non-invasive continuous positive-pressure ventilation. These infants frequently exhibit bowel dysmotility with low-grade bilious aspirates while they are establishing full enteral milk feeds (4). Clinical suspicion of serious intestinal conditions such as NEC leads to the cessation of enteral feeds, often with the use of intravenous antibiotics, allowing for more clinical evidence to guide clinical management. Thus, accurate assessment and reassurance of a normal bowel sound being heard is a surrogate marker of a healthy and viable gut and contributes to the early detection of bowel pathology. It is important to note that assessment of the abdomen is multimodal, and beyond the bowel auscultation for bowel peristalsis, a thorough clinical examination of the abdomen is vital.

Although the traditional method of bowel auscultation is easy to perform and provides a qualitative description of the presence or absence of bowel sounds, it has several limitations (2). Even full 3-min auscultations in a 24-h period reflect <1% of the 24-h period assessed for peristalsis. It lacks precision due to subjective reporting; lacks reproducibility; relies on the human ear to perceive transmitted sounds with potential noise interferences in a neonatal intensive care environment; relies on the quality of the stethoscope used for detection; and relies on prior operator experience in this skill, which influences reporting (1–5). Bowel sounds as a marker of peristalsis and bowel health are linear time assessments, and, like heart rate variability, non-linear kinetics are not currently detected. We speculate that non-linear kinetics may offer early pre-clinical detection of sepsis and or NEC (6, 7).

Thus, quantitatively detecting bowel sounds would lead to more accurate clinical decision-making for the early detection of diseased bowel states. No bowel sounds heard on abdominal auscultation is an ominous clinical sign (8). We postulate that continuous monitoring of neonatal bowel sounds quantitatively might have clinical benefit in extremely preterm infants and in high-risk term infants at risk of bowel dysfunction.

The traditional method of bowel sound detection (using a stethoscope) can be extrapolated to obtain electronic waveforms (using an electronic stethoscope), leading to increased objectivity in analyzing bowel sounds (9, 10). Another objective or direct method of assessing bowel motility is to visualize bowel movements in real time using US (11). A high-frequency Linear US probe placed over the abdominal cavity can detect true bowel motility, seen as worm-like or circumferential opening and closing of bowel segments in real time (11, 12).

Bowel peristalsis is affected by several gastrointestinal pathologies. There is a loss of bowel motility in segments affected by NEC, and bowel segments proximal to non-inflammatory obstructive pathologies demonstrate hyperperistalsis (12, 13). Recent publications by pediatric radiologists have proven the role of point-of-care abdominal US in diagnosing NEC and the role of bowel peristalsis in the diagnostic matrix (12–15). There are no data on the characterization of normal bowel motility in healthy preterm and term neonates as they progress through achieving full enteral feeds. There is emerging evidence that point-of-care bowel US in the hands of the treating neonatologists adds to the diagnostic accuracy in assessing clinically suspected bowel pathologies (12). To address this identified knowledge gap, further research is required on what constitutes normal bowel motility in healthy preterm and term infants.

The study's aim was to determine the value of conventional bowel sound auscultation using two different methods simultaneously. We compared bowel peristalsis detected using acoustic recordings from an electronic stethoscope with bowel peristalsis detected using US in neonates with no known bowel disease on full enteral feeds.

## Methods

This is a prospective observational cohort study in preterm and term neonates on full enteral feeds with no known bowel disease. A 3M™ Littmann® Model 3200 electronic stethoscope was used to obtain a continuous 60-s recording of the bowel sounds, including manual count and simultaneous computerized digital recordings. We used the diaphragm mode, which amplifies the sounds from 20 to 2000 Hz but emphasizes the sounds between 100 and 500 Hz. It samples at 125 Hz and has digital electronic filtering for ambient noise reduction.

**Figure 1:**
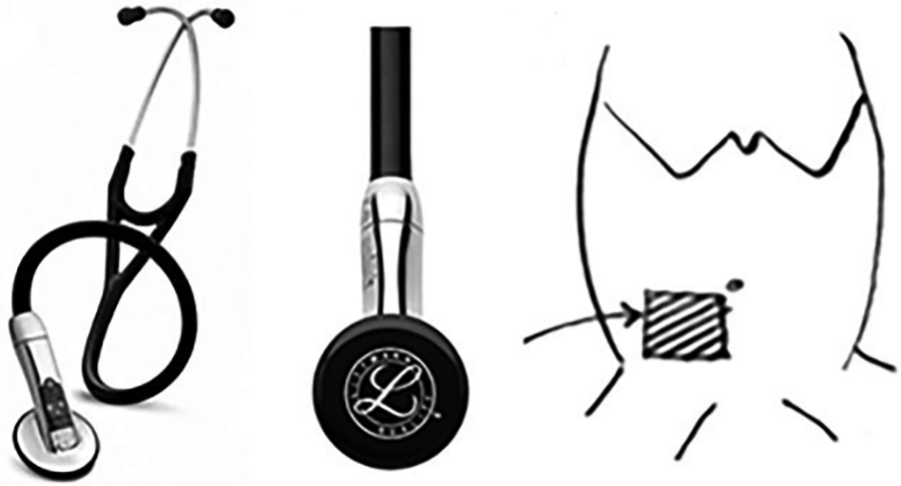
(**A**,**B**) Images of the 3M™ Littmann® model 3200 electronic stethoscope used to obtain a continuous 60-s recording of the bowel sounds. (**C**) The stethoscope acquisition region: right iliac fossa beneath the umbilicus.

**Figure 2:**
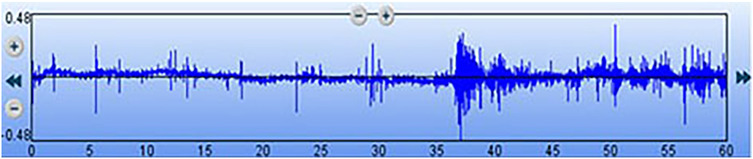
Digital waveform recording of the bowel sounds obtained using a 3M™ Littmann® model 3200 electronic stethoscope. The x-axis represents the time elapsed (total of 60 s), and the y-axis indicates the relative sound amplitude.

The digital stethoscope was connected via Bluetooth to the laptop computer to transmit the de-identified waveform file for advanced signal analysis. While the first investigator placed the diaphragm of the digital stethoscope (measuring 3 cm), a second investigator blinded to the signals from the stethoscope concurrently placed a high-frequency 12-Linear US probe adjacent to the diaphragm over the set region matching time duration, to visually assess bowel motility. The commencement of the two recordings was synchronized with the manual tap of the stethoscope diaphragm onto the US probe. The region chosen was the iliac fossa beneath the umbilicus (Figure 1C). A total of 30 recordings were collected each for the acoustic recording using the electronic stethoscope (Audio P) and the US bowel motility recordings (Ultrasound P). Each of these recordings was analyzed for the sound duration in seconds and represented as a percentage of bowel peristalsis detected in the total 60 s of recording. The stethoscope and the US probe were placed by two independent investigators blinded to each other's recording of observations. We used segmentation using start and end times from the annotation file for both the audio and the visual files for each of the subjects. We determined the bowel or non-bowel sound and peristalsis movement periods by annotating the start and the end times to ascertain the time segments for comparison (Figure 2). The annotations were performed independently at a later date on de-identified data sets by the author AP for both the audio and the video samples randomly and cross-validated with author MT.

The studies were done between 30 min and 120 min after the enteral milk feed was administered.

The study period included 1 January 2019–31 August 2019. The eligibility for the study included healthy preterm and term infants not requiring invasive ventilation; preterm neonates receiving continuous positive-pressure airway pressure (CPAP) as a form of respiratory support were included in this study. The study was performed in the pre-prandial period on all participants, and they received >50% of their full enteral (150 ml/kg/day) milk feed requirement. Infants with significant chromosomal anomalies and with known gastrointestinal congenital abnormalities were not included in the study. None of these neonates were hemodynamically unstable.

Written consent was obtained from the parent or guardian of each participant. Data were extracted from the 3M™ Littmann® Model 3200 electronic stethoscope and the GE Healthcare Vivid E95 US machine system and analyzed using Stata MP12 (V.17 MP, Stata Corp, College Station, Texas, USA). The peristalsis time was not normally distributed and could not be statistically transformed to normalize. Thus, non-normally distributed data were tested with Wilcoxon paired rank–sum statistical analysis. Estimates of median differences and 95% CIs were obtained using bootstrapped quantile regression with 250 repetitions and the threshold for *p* of 0.05 was considered statistically significant. This study was approved by the Sydney West Area Health Service Human Research and Ethics Committee.

## Results

The study population ages ranged from 27 to 43 weeks including preterm and term neonates with birth weights ranging from 580 to 4,600 g, and all study participants were on >75 ml/kg/day of their total enteral milk feed (Table 1).

**Table 1:** Demographic profile.

Patient profile	Study infants (*n* = 30)
Gestational age at time of study, weeks [median (interquartile range)]	33 (27–43)
Sex (male/female)	19/11 (63.3%/36.6%)
Birth weight, g [median (interquartile range)]	1,892 (580–4,600)
On enteral milk feeds >75 ml/kg/day	30 (100%)

**Figure 3:**
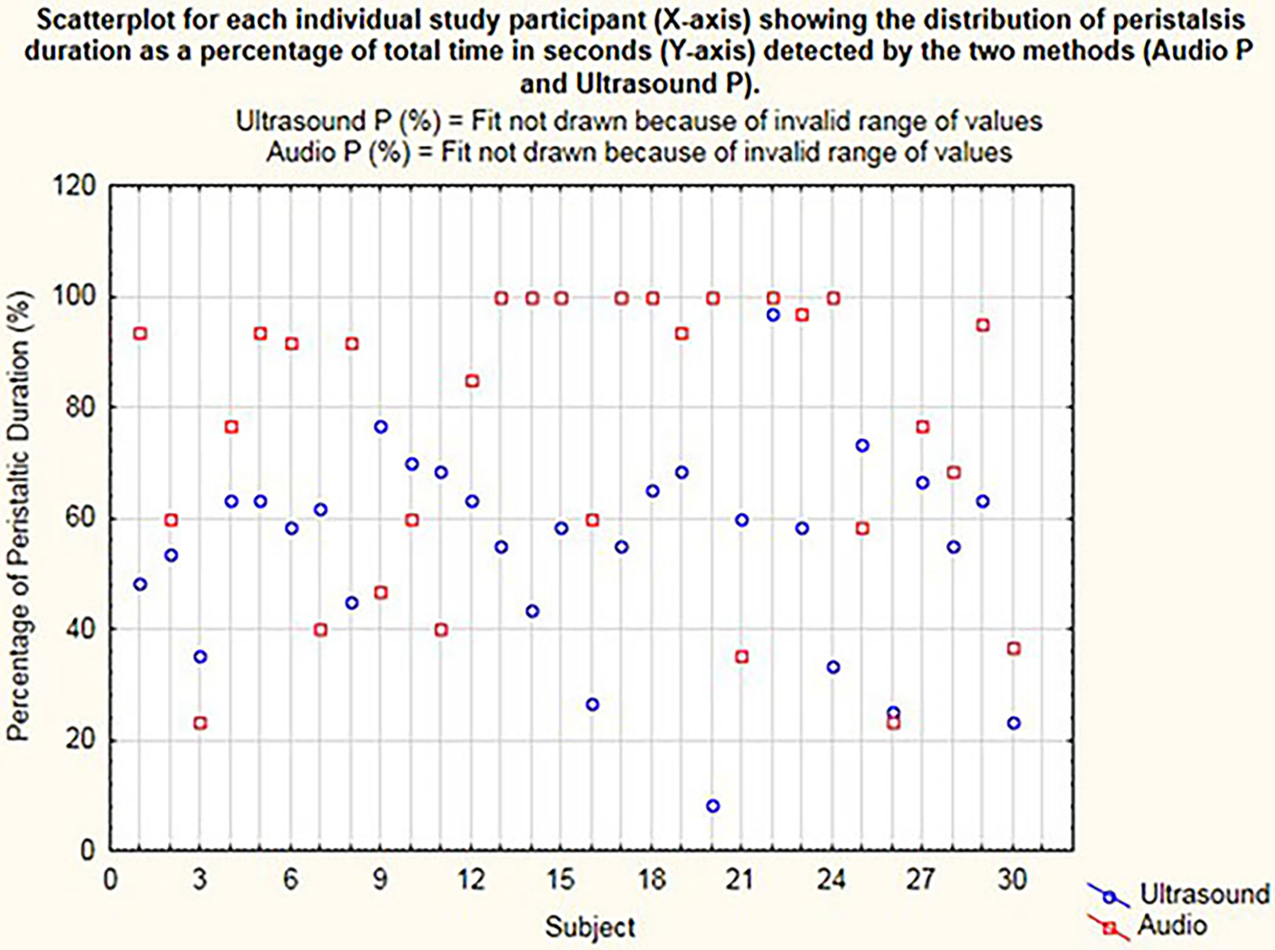
Dot plot for each individual study participant (x-axis) showing the distribution of peristalsis (time in seconds) detected by the two methods (audio P and ultrasound P).

On the dot plot (Figure 3), the x-axis represents the individual participants. Each dot on the y-axis represents the total time during which the peristalsis was detected as a percentage calculated from the total 60 s of recording time, concurrently with the stethoscope and US methods. This is represented as Audio P for the digital waveform recordings from the electronic stethoscope and as Ultrasound P for the US detection of bowel motility.

**Figure 4:**
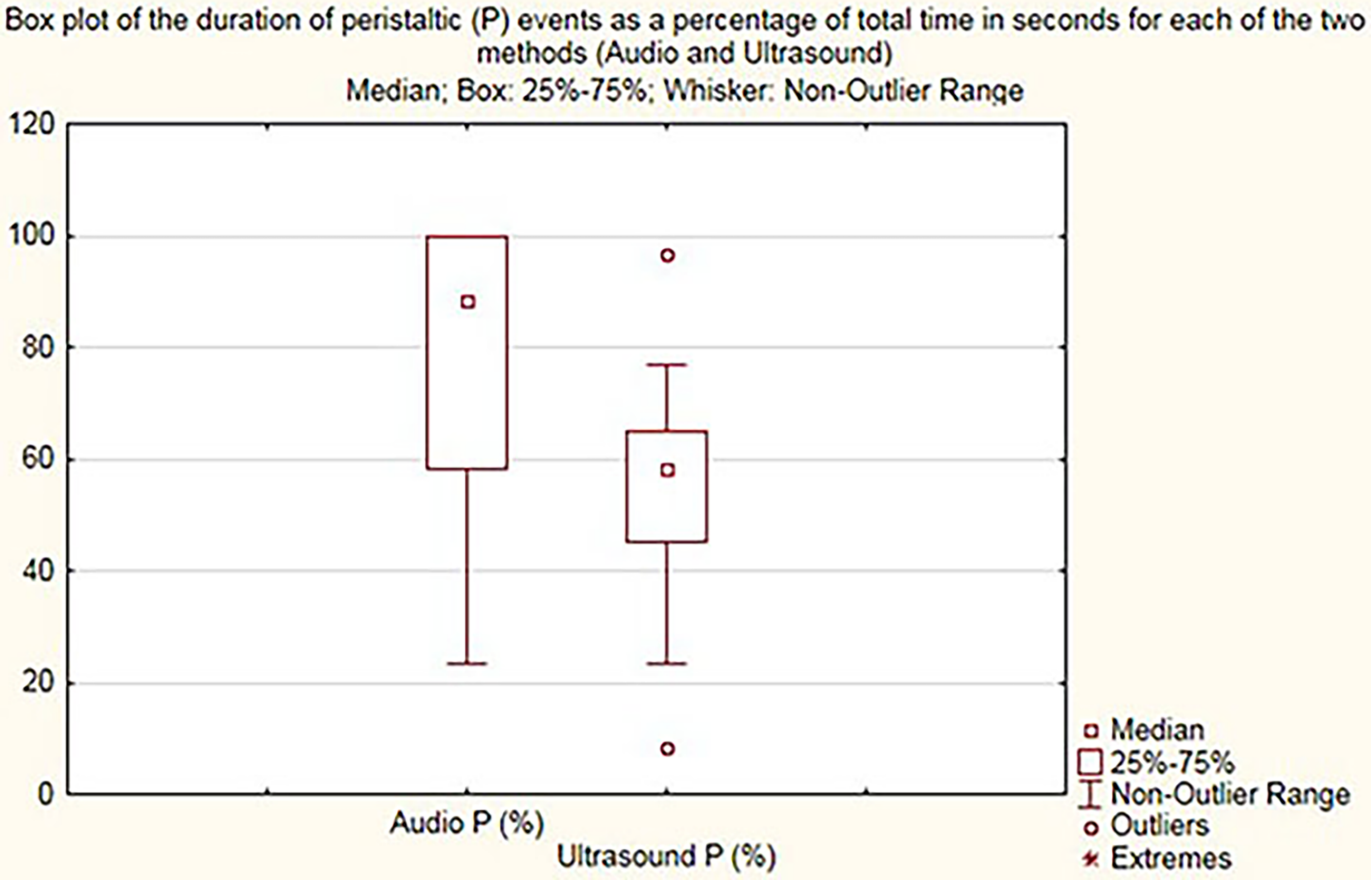
Box plot demonstrating peristalsis duration (percentage) for audio (audio P) and US (ultrasound P).

The Audio P and Ultrasound P box plot shows the summary of peristaltic percentages in the 30 cases for audio samples and US samples, respectively (Figure 4). The calculation Audio P—Ultrasound P is the difference in Audio P and Ultrasound P for the peristaltic percentages summary of all 30 samples.

Peristalsis time was examined for distribution and found to be not normally distributed (Figure 3). Transformation of these data did not resolve the non-normalization of the data, so a non-parametric analysis was applied with the Wilcoxon rank–sum (Mann–Whitney) test. A 0.5 median quantile regression analysis using boot-strapped repetitions (*n* = 250) was used to examine differences in median peristalsis times.

On comparing the times of bowel sound detection by digital stethoscope recording to times of visual detection of bowel movements upon use of US, the median time of peristalsis with US was 58% compared to 88.3% with acoustic assessment (*p *< 0.002), with regression difference in medians of 26.7% with 95% CI 5%–48%.

## Discussion

Our study demonstrates disconcordance between auscultated bowel sounds and visualized bowel motility upon use of US within the set region of the neonatal abdomen. In 22 participants, bowel sounds when heard on auscultation did not correspond to visible bowel movement detection upon the use of US concurrently in the same set region. Direct visual observation of bowel movement is an accurate assessment of bowel peristalsis (12, 15, 16). This allows for the investigation of auscultated bowel sounds that were found to be over-estimated in our study. The findings of our study showing poor concordance between real-time US detection and auscultation question the accuracy of bowel sound auscultation for bowel pathologies particularly affecting localized bowel segments.

The US probe placed over the abdominal wall has a small linear footprint, which allows for the interrogation of bowel segments within a small, localized area over the abdomen. However, bowel sounds are more readily transmissible across the abdominal cavity, resulting in an overestimation of the events by auscultation, as shown in our study. These events represent transmitted bowel sounds occurring in healthy bowel segments in proximity and are not necessarily representative of the site of interrogation. Localized bowel pathology in neonates such as non-inflammatory obstruction results in hyperperistalsis in the proximal segments surrounding the site of obstructed bowel with no or poor peristalsis detected in the region of the pathology (12). Using a standard assessment tool such as a stethoscope to assess bowel movements in a region of interest would thus still pick up the transmitted bowel sounds, leading to an inaccurate assessment. The direct visualization of bowel motility is an objective way to determine bowel movements in real time. This is described by Faingold et al. (15), who, in assessing bowel movements in preterm and term infants between 27 and 41 weeks, classified bowel motility as normal if peristalsis was detected between 1 and 10 times during a 60-s period of US recording. A broad range of analytical approaches has been used in bowel sound analysis in adults. This is the first clinical research on bowel sounds in neonates using both automated bowel sound analysis and point-of-care US.

It is likely that subtypes of bowel sounds exist and potentially have a broad dynamic range. Further research into the characterization of these bowel sounds as a combination of automated bowel sound analysis (bowel sound recording and analysis of the waveform) and real-time visualization of bowel motility using US has the potential for clinical application in assessing neonates with feed intolerance and early detection of diseased bowel state such as NEC. Furthermore, intestinal injury can be prevented by early cessation of enteral feeds with a timely diagnostic approach. Detection of normal bowel peristalsis can be reassuring in suspected cases of CPAP belly syndrome, a common clinical scenario when continuous positive-pressure ventilatory respiratory support is used and feed intolerance occurs, particularly in extremely preterm infants (4).

All recordings were from a Littmann 3,200 digital stethoscope for a 60-s total duration for each of the recordings, from each participant. However, there were several limitations. The sample size was small, and the duration of the bowel sound assessments was limited. A time of 60 s is relatively short but reflects what can realistically be achieved in clinical practice. Most babies have normal spontaneous rapid body movements affecting their trunks, and our study participants were otherwise all healthy babies. Thus, to maintain a reliable trace output, one minute was chosen as an acceptable time to record bowel sounds to allow for no displacement of the stethoscope or the US probe from the set region over the abdomen. Also, in practice, neonatal clinicians would not spend more than 60 s at the bedside to record bowel peristalsis clinically, using a stethoscope, in a busy neonatal care environment. There was only one recording each for both methods for each infant that participated in the study. While the two investigators were blinded to each other's findings, the final analysis of the duration of bowel sounds was determined by a single operator with experience in neonatal bowel US assessments. Lastly, this study has assessed the duration of the bowel sound assessment, but it does not assess the subtypes or characterize bowel sounds into descriptions of hyperactive, hypoactive, or absent.

Thus, our study is the first study in neonates designed to assess the reliability of using a stethoscope to determine bowel sound activity that guides clinical assessment of their gastrointestinal tract. Further research is necessary into the application of artificial intelligence and the development of dedicated bowel sound devices integrating the real-time US detection of bowel motility and multi-modal sensors for automated bowel sound detection. This could lead to continuous bowel sound monitoring as a surveillance screen for early detection of diseased bowel states.

## Data Availability

The raw data supporting the conclusions of this article will be made available by the authors, without undue reservation.
